# Use self-construction theory to understand Daka destination information sources and motivation impact on tourism intention

**DOI:** 10.3389/fpsyg.2022.928331

**Published:** 2022-08-25

**Authors:** Yitao Chen, Junwei Zhao, Jianyi Ding, Lei Wang, Shengjun Yuan

**Affiliations:** ^1^Management School, Hainan University, Haikou, China; ^2^School of Business, Guilin University of Electronic Technology, Guilin, China; ^3^School of Business, Chungbuk National University, Cheongju, South Korea

**Keywords:** tourism information source, tourism motivation, self-construction, tourism intention, Daka destinations

## Abstract

Daka destinations refer to tagging one’s visit to a popular destination by posting on social media. As a novel tourism concept derived from digital media in the post-pandemic era, Daka destinations have become a major option for potential tourists; thus, investigating tourist intentions toward them is of utmost significance to tourism recovery. Based on the viewpoints of information sources of Daka destinations, tourism motivations, and self-construction, this study investigates the research framework of potential tourism intentions through three scenarios. The findings revealed the following: (i) Different information sources have different stimuli for potential tourists, and WeChat Moments exerted a positive impact on tourism intention because of credibility; tourism bloggers from Weibo exert a more significant positive impact on tourism intentions of potential tourists because of professionalism. (ii) Considering the credibility of WeChat Moments, the extrinsic motivation of potential tourists exerted a more significant impact on tourism intentions; regarding professional tourism bloggers, the intrinsic motivation of potential tourists exerted a more significant impact on tourism intention. (iii) Regarding the credibility of WeChat Moments, dependent self-construction potential tourists with extrinsic motivation exerted a more significant impact on tourism intention. Regarding tourism bloggers with high professionalism, independent self-construal potential tourists with intrinsic motivation exerted a more significant impact on tourism intention. This study enriches the research mechanism of the formation path of potential tourists’ tourism intention, extends the self-construction theory to the research field of using social media to collect Daka destinations tourism information, and provides a reference to subsequent research on potential tourists’ tourism intention.

## Introduction

With the development of network information technology, consumers’ lives are covered on TV, radio, newspapers, computers, mobile phones, and other media. Tourism experience sharing process, communication mechanism, and consumption mode are changing. The Internet has become an important information channel for obtaining tourism information and selecting destinations (Morosan and Jeong, 2008). The availability of social media platforms and technologies has enabled visitors to increasingly digitize and share online knowledge and information (Buhalis and Law, 2008; Volo, 2010) as well as emotional and experiential moments (Jacobsen and Munar, 2012). Consumers’ cognition of the world not only comes from their own direct experience, but also depends on the information spread by various media. Consumers can share and collect tourism information on social media, which has diversified the ways consumers receive tourism information. Social media has become an important source of information for consumers before travel (Luo et al., 2005).

Daka destinations are a novel tourism concept derived from digital media in the post-pandemic era, with a core trait of its social flow, which has become a key choice for potential tourists. Daka destinations are regarded as going deep into the destination and enjoying the real local experience. It has quickly become an indispensable part of the tourist wish list and is the latest trend to promote tourism (Connell et al., 2021). The biggest difference between Daka destinations and traditional tourism attractions is that potential tourists in the former obtain information on tourism attractions through the network media, combined with the tourists’ values and interests, motivating them to generate tourism. While Internet celebrity culture is sweeping social media, giving tourism attractions the color of Internet celebrity signifies a stronger link between tourism attractions and users (Tran and Strutton, 2014). From the communication perspective, Daka destinations are not planned or constructed but created by the construction of media and the continuous spread of tourists. The concept of Internet celebrity has ushered in new communication power to traditional tourism, spawning numerous phenomenal Daka destinations. For example, although Lugu Lake and Meili Snow Mountain in Lijiang, Yunnan, Yangshuo West Street, and Longsheng Terraces in Guilin, Guangxi, Jiuzhaigou in Sichuan, and The Ends of the Earth in Hainan are old celebrity tourism attractions, owing to their attributes and multiple dissemination, they have sprung up in several tourist attractions with different characteristics, and their popularity in holidays is far more than regular tourism attractions. Daka destinations have not only become iconic content that attracts traffic and are a must for mass tourism but also become an integral part of the city’s image to some extent.

The research on tourism information in academia primarily focuses on the significance of information (Bieger and Laesser, 2004), audience and generation stage of information (Ortega and Rodríguez, 2007), and dissemination medium of information (Gao, 2003). Regarding information sources, it is rather crucial for tourists to obtain reliable relevant tourism information (Litvin et al., 2008). Meanwhile, the source of tourism information affects tourism decision-making, and the quality of tourism information sources affects the decision-making intention of tourists (Meng et al., 2021). Social media has evolved into one of the important sources of information for tourism, and it is suggested that further extensive research and investigation of its impact on tourism is needed in the academic community (Leung et al., 2013; Cohen et al., 2014). In addition, investigating the potential tourists’ tourism intention to Daka destinations from the standpoint of tourism motivation and self-construction can better elucidate the formation and dissemination mechanism of Daka destinations. The motivation theory is a key factor affecting potential tourists’ intention to travel to Internet celebrity attractions. Thus, dividing tourism motivation into intrinsic motivation and extrinsic motivation has the advantages of applicability, diagnostics, and operability for the research of Internet celebrity tourist attractions (Park and Nicolau, 2015). The development of Daka destinations is inseparable from the dissemination and promotion of tourism information by social media, and self-construction can estimate the use of social media platforms (Chu et al., 2015). In social media dissemination of online celebrity tourist attractions, does self-construction affect potential tourists’ adoption of tourism information sources (moments vs. travel bloggers)? Hence, it is imperative to examine the impact of Daka destination information source and tourism motivation of potential tourists on tourism intention from different viewpoints, which is of utmost significance for tourism recovery in the post-pandemic era.

Based on the two representative dimension attributes of credibility and professionalism of Daka destination information source features, under the context of different tourism motivations and self-construction, this study tests the impact of Daka destination information source on potential tourists’ tourism intention through three experimental scenarios. This study attempts to address the following three questions: (i) With different Daka destination information sources, are there differences in impacts on potential tourists’ tourism intention? (ii) In the stimulation of tourism intention by information source, is there a moderating effect on tourism motivation of potential tourists? (iii) In the stimulation of tourism intention by information sources, is there a moderating effect on the self-construction of potential tourists? This study provides a theoretical basis for creating and disseminating Daka destinations and provides references for developing new tourism in the context of digital media in the post-pandemic era.

## Theoretical analysis and hypotheses

Based on the credibility and professionalism of the characteristics of Daka destination information sources, this study examines the impact of Daka destination information sources on potential tourists’ tourism intentions under the background of different tourism motivations and self-construction.

### Impact of information source on potential tourists’ tourism intention

Potential tourists’ tourism intention is a subjective judgment of potential tourists’ possible behavior decisions in future. When the necessary conditions are met, they will be transformed the behavior into actual behaviors, such as information collection, behavior decision-making, and information sharing (Han et al., 2021). Information source plays a vital role in the selection process of tourism destination (Beerli and Martin, 2004). For example, by simulating tourists’ online search for nine tourism destination place name keywords, it is found that most of the search results come from social media, which shows the importance of social media as a channel for tourism information dissemination (Xiang and Gretzel, 2010). As more and more tourists share their travel experiences with social media, social media has become an important information source and decision reference for tourists’ tourism decisions (Luo and Zhong, 2015). Reportedly, insights, experiences, and opinions shared by users on social media have high reliability and credibility (Dickinger, 2011). The professionalism and relationship strength of social media information publishers can affect tourism behavior intention (Lyu et al., 2018). Of note, information source credibility depends on the information receiver’s judgment of the information disseminator, and the information disseminator should have profound knowledge, colorful experience, and new authenticity and possibility (Belch and Belch, 2004). Moreover, information credibility stems from the subjective and objective judgments of the audience, in which the object of subjective judgment is the credibility, professionalism, and attractiveness of the information source, and the object of objective judgment is the quality and accuracy of the information content (Metzger et al., 2003). Expertise is often used as an indicator of the ability to provide relevant information (Feick and Price, 1987). The professionalism of the information source will determine the influence and authority of the information. The professional information source is easier for the receiver to believe and make decisions (Gilly et al., 1998).

As per the information source credibility theory, the content published on social media comes from different channels, and when the information source is from a credible channel, it enhances the persuasion of individuals (Hautz et al., 2014). According to the identity of the information publisher, information sources can be categorized into professional sources and private sources. Consumers have different opinions on the credibility of professional sources and private sources. Indeed, professional sources are more credible, while private sources are only a part of the information search process (Cox et al., 2009). Information professionalism is the information receiver’s perception of the level of professional knowledge and experience of the information dissemination source. Information credibility is the information receiver’s perception of the reliability and security of the information source. Both professionalism and credibility exert positive impacts on subsequent behaviors (Chao et al., 2005). Reportedly, professionalism and usefulness increase individuals’ willingness to share information (Jeong and Lambert, 2001), credibility affects individual attitudes (McGinnies and Ward, 1980), and professionalism affects individuals’ willingness to buy (Gilly et al., 1998). Hence, the following hypotheses are proposed:

H1: The higher the information source credibility in WeChat Moments, the higher the potential tourists’ tourism intention to Daka destinations.

H2: The higher the information source professionalism of tourism bloggers from Weibo, the higher the potential tourists’ tourism intention to Daka destinations.

### Impact of tourism motivation of potential tourists on tourism intention

As a major concept in the research of Daka destinations, tourism motivation is a crucial precondition to revealing potential tourists’ pursuit of Daka destinations (Ye et al., 2021). Elucidating the correlation between tourism motivation and tourism intention is of great significance for comprehending the characteristics and destination management of Daka destinations. Tourism motivation is the psychosocial force that predisposes individuals to choose and participate in tourism activities, and motivation positively affects the behavioral intentions and decision-making of tourists (Huang and Hsu, 2009). Potential tourists with intrinsic motivation tend to focus on intrinsic rewards, self-improvement, self-direction, and the pursuit of inner satisfaction and enjoyment. Nevertheless, potential tourists with extrinsic motivation tend to divert their attention from self-direction and constrain their response to their intrinsic needs, focusing more on extrinsic rewards (Liu et al., 2021). Tourism motivation is an integral part of tourism behavior research, which affects the image of the destination (Beerli and Martì, 2004), cognition and emotion (Wang and Qu, 2013), and the overall image of the destination (Wang et al., 2019). This study explores the impact on tourism intention based on different information sources and based on intrinsic motivation and extrinsic motivation in self-determination theory. Intrinsic motivation is a key factor of behavioral willingness, including a desire to escape, interest, prestige, health, risk-taking, and self-realization (Zhou et al., 2021). Based on research in social media, extrinsic motivation comprises relationship building, prestige gain, reciprocity, material rewards, and other additional benefits (Wang et al., 2017). Extrinsic motivation correlates with the perception of the quality of information obtained from social media, while intrinsic motivation correlates with pleasure and intrinsic emotions when using social media platforms (Sussman and Siegal, 2003).

In the virtual network environment, consumers are more inclined to accept the information released by the surrounding people, while WeChat is mostly based on the social circle of acquaintances in real society, so the information acceptance in WeChat is based on trust (Lyu et al., 2018). Compared with other social media, a large number of content producers on Weibo are professional profit-making organizations, video experts, and online celebrities. They are professionals in tourism experience, tourism information mastery, and tourism video production. Highly professional cognition of information sources will enhance intrinsic motivation (Cusella, 1982). According to the social information processing theory, if potential tourists feel that the information is more compatible with themselves, they will have a sense of correctness and the persuasive effect of the information will be stronger (Salanick and Pfeffer, 1978). Thus, it is believed that under the stimulation of different information sources, the tourism motivation of potential tourists differs, and the impact on tourism intention varies as well. Hence, the following hypotheses are proposed:

H3: Under the stimulation situation of information sources from WeChat Moments, potential tourists’ tourism intention based on extrinsic motivation is higher.

H4: Under the stimulation situation of information sources from Weibo travel bloggers, potential tourists’ tourism intention based on intrinsic motivation is higher.

### Moderating effect of self-construction of potential tourists

Self-construction refers to how a person views the relationship between self and others and society (Markus and Kitayama, 1991), whether their thoughts, emotions, and actions focus on the relationship between themselves and others or the difference between themselves and others (Singelis, 1994). Thus, self-construction can be divided into two types: independent self-construction and dependent self-construction. In a social media interaction, the self-construction orientation of users positively modulates the connection between platform use intention and engagement (Hu et al., 2016). The nature of the social media context of socialization relies on the sense of belonging, and social connection needs to be entrenched in the interdependence of users. Individuals with dependent self-construction orientations exhibit extra pronounced attitudes than individuals with independent self-construction orientations and behavioral changes, as individuals with dependent self-construction orientations are more susceptible to environmental factors (Sia et al., 2009). Moreover, individuals with an independent self-construction orientation follow their inner choices in the travel process, focus on their preferences and interests, but not pay attention to others’ feelings or consider the opinions of reference groups (Li, 2018). While individuals with independent self-construction are self-centered and have weak social connections, those with dependent self-construction have a rather close interpersonal network and strong social support relationships (Na et al., 2014). Some previous studies examine different impacts of self-construction orientation on individual behavior from the viewpoints of motivation and cognition. Individuals with an independent self-construction orientation tend to be motivated by intrinsic motivation and self-satisfaction, looking for information that matches themselves, whereas those with a dependent self-construction orientation are more inclined to be motivated by extrinsic motivation, endeavor to adapt, and belong to the corresponding social group, thereby building a relationship with the group (Holbrook and Hirschman, 1982). Independent self-construction tends to make subjective judgments based on self-perception and satisfy their internal needs (Hong and Chan, 2015). Dependent self-construction pays more attention to their interpersonal interactions with others and, at the same time, improves their sense of belonging and satisfies their own motivational needs through reciprocal behaviors (Na et al., 2014). Therefore, this paper believes that independent self-construction pays more attention to intrinsic motivation in the process of checking the tourist information source. In comparison, dependent self-construction pays more attention to extrinsic motivation. Hence, the following hypotheses are proposed:

H5: In the context of stimulation by WeChat Moments information source, impacts of potential tourists with extrinsic motivation on tourism intention are regulated by dependent self-construction.

H6: In the context of stimulation by tourism bloggers from Weibo information source, the impacts of potential tourists with intrinsic motivation on tourism intention are regulated by independent self-construction.

## Experimental materials and research methods

The following is the case site selection and research method.

### Case sites selection

In this study, Zhangzha Town, Jiuzhaigou County, and northwest Sichuan were selected as case sites. The main Daka destinations are Jiuzhaigou tourism attraction, Eternal Love tourism attraction, and Ganhaizi. Besides, there are many lakes and forests, with abundant animal and plant resources, such as giant pandas—well-known in the ranking of Daka destinations in China. Of these, Jiuzhaigou holds multiple titles such as a world natural heritage, national key scenic spot, national AAAAA tourism attraction of China, national natural reserve, national geopark, and world network of biosphere reserves. In the post-pandemic era, to open up and develop tourism orderly and safely, Sichuan Province held a restoration and opening ceremony of Jiuzhaigou tourism attractions on 28 September 2021.

### Experimental materials and research methods

In this study, the tourism information sources of Daka destinations in Jiuzhaigou were presented through WeChat Moments (information sender and information receiver are related in some way and are familiar with each other) and Weibo (information sender and information receiver are not related in some way, and they do not know each other) to different subjects, and the text description and pictures in the information display content are identical^1^. For the validity of the questionnaire, first, an attention detection item was added, and the invalid questionnaire was filtered by detecting whether the person filling in the questionnaire could judge the difference between the information source channel. Besides, to eliminate the influence of travel experience on the tourism intention of subjects, whether they have been to tourist attractions was first asked, and then they were asked to read the test stimulation materials and fill in the questionnaire according to their opinion. Of note, responses were scored using a seven-point Likert scale (1 = totally disagree and 7 = strongly agree).

Taking Chinese consumers as the research object, 1,042 valid sample data were collected in September 2021, with an effective rate of 87.4%. From the data obtained from the sample gender, it can be seen that the male-to-female sex ratio is nearly 1:1. The age distribution of the data is quite special. People that account for 89.3% of the total number of tested samples are concentrated on people under 40 years old. The reason is that people in this age group belong to the Internet active people and are more willing to cooperate with the experiment. The Cronbach’s α values of the experimental variables were all above 0.85, the KMO values were all above 0.7, and the values of Bartlett’s spherical test were all 0.000, indicating that the reliability and validity of the experimental design of the questionnaire were high.

## Research design and hypothesis verification

This section will show three progressive experimental studies in turn. Experiment A explores the influence of different information sources on the travel intention of potential tourists based on the different characteristics of the information sources. Then, the influence of the interaction between different information sources and different tourism motives on the tourism intention of potential tourists is further explored through experiment B. Finally, in Experiment C, we examined the self-construction factors of potential tourists to consider the interaction relationship of several factors.

### Experiment A

Experiment A aimed to provide an initial test of hypotheses 1 and 2. We predicted that different information sources have different stimuli for potential tourists. WeChat Moments would exert a positive impact on tourism intention because of credibility; tourism bloggers from Weibo would exert a more significant positive impact on the tourism intentions of potential tourists because of professionalism.

#### Pre-experiment test

To identify the social media of Daka destination information source, we randomly enrolled 46 subjects (male 52.08%; median age = 25.61 years, SD 2.33) by Wenjuanxing for pre-testing. During the pre-test, 10 groups of popular social media platforms with the same tourism information were presented to subjects, who were asked to indicate their most commonly used media platforms, their attitudes toward each social media platform, and their willingness to continue using them. Of note, the measurement items were referred to as the mature scale (Zhang et al., 2019), and relevant modifications were made depending on the situation. The attitude scale items were “I feel happy during the process of using this social media platform” and “I am interested during the process of using the social media platform.” The willingness scale for continuing to use was “this social media platform deserves my attention and use,” and “I will continue to pay attention to and use this social media platform in future.” Accordingly, WeChat (WeChat is generally a social tool between relatives and friends. Users can always post text and pictures through WeChat Moments) and Weibo (Weibo is generally a social tool between strangers, which can understand the hot topics) were set as the social media platform of the information source. Of note, all subjects used WeChat and Weibo, and they generally exhibited a positive attitude toward WeChat and Weibo (*M* = 5.88, SD 1.11) and later continued to use (*M* = 5.44, SD 1.20). Moreover, WeChat and Weibo as information sources displayed significant differences between groups [*M*_WeChat_ = 4.67, *M*_Weibo_ = 5.39, *F*_(1,44)_ = 22.14, *P* < 0.001], and the experimental materials fulfilled the requirements of the experimental operation.

#### Research design

In Experiment A, 2(information source: WeChat Moments vs. tourism bloggers from Weibo) × 2(source features: credibility vs. professionalism) research design was adopted, and we examined the impact of Daka destination information source on potential tourists’ tourism intention. The credibility and professionalism scales of Daka destination information source were based on (Meng et al., 2021), and relevant modifications were made depending on the situation. The tourism intention scale was based on Wu and Huang (2019). In all scales, we adopted a seven-point Likert scale (1 = disagree at all and 7 = strongly agree). Subjects were asked to fill in the demographic information at the end of the questionnaire. The experiment was designed with attention verification and continuous extreme value/continuous equal value exclusion screening rules. Finally, we obtained 166 valid samples (male 50.8%; *M*_age_ = 29.71, SD 6.77). Of these, the Daka destination information source in WeChat Moments was the information published by the researcher in WeChat Moments. In addition, the researcher’s WeChat friends were taken as the experimental objects, and 80 subjects were collected for testing. Furthermore, the Daka destination information of tourism bloggers from Weibo was tested on Wenjuanxing with 86 random subjects.

#### Results

**FIGURE 1:**
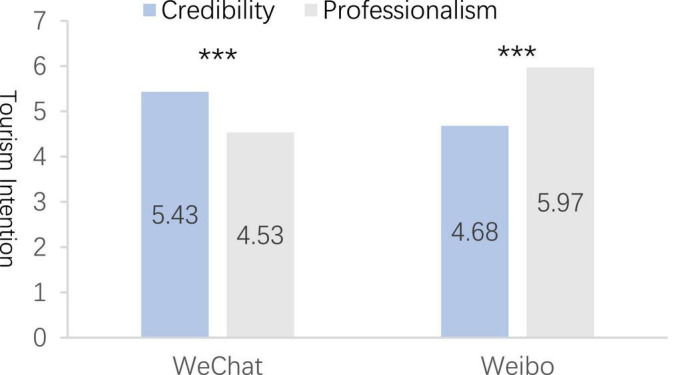
The influence of information source types of internet celebrity tourism attractions on the tourism intention of potential tourists. ^***^p < 0.01.

The between-group analysis of potential tourists’ tourism intention by the social media platform information source revealed that WeChat Moments and tourism bloggers from Weibo as the information source significantly differed between groups [*M*_WeChat Moments_ = 4.78, *M*_tourism bloggers from Weibo_ = 5.63, *F*_(1,164)_ = 12.47, *P* < 0.001], and the experimental manipulation was successful. In addition, two-way analysis of variance (ANOVA) results (Figure 1) suggested the principal effect analysis (PEA) of information source on potential tourists’ tourism intention (α = 0.872) was significant [*F*_(1,164)_ = 12.47, *P* < 0.001]. The features of information source exerted a significant impact on the PEA result of potential tourists’ tourism intention [*F*_(1,164)_ = 22.77, *P* < 0.001], and their interaction effect was significant on the PEA result of potential tourists’ tourism intention [*F*_(1,164)_ = 17.54, *P* < 0.001]. In addition, the credibility of WeChat Moments as an information source (α = 0.911) and professionalism (α = 0.901) differed significantly [*M*_credibility_ = 5.43, *M*_professionalism_ = 4.53, *F*_(1,164)_ = 27.40, *P* < 0.001]. Furthermore, the credibility of tourism bloggers from Weibo as an information source (α = 0.895) differed significantly from that of professionalism (α = 0.899) [*M*_credibility_ = 4.68, *M*_professionalism_ = 5.97, *F*_(1,164)_ = 18.99, *P* < 0.001]. Hence, H1 and H2 are confirmed.

#### Discussion

Experiment A, based on the credibility and professionalism of previous research information source, demonstrated that different types of social media as tourism information sources have different effects on potential tourists’ tourism intentions. Experiment A demonstrated that the credibility of WeChat Moments as an information source exerted more significant positive impacts on potential tourists’ tourism intention than professionalism, and tourism bloggers from Weibo as an information source of professionalism exerted more significant positive impacts on potential tourists’ tourism intention than credibility. The stimulation of the Daka destination information source of WeChat Moments to potential tourists’ tourism intention was based on the fact that WeChat Moments had higher credibility. In addition, the credibility of Daka destinations on WeChat Moments was the key factor for potential tourists to generate tourism intention. Moreover, the stimulation of potential tourists’ tourism intention by the Daka destination information source of tourism bloggers from Weibo was based on the fact that tourism bloggers from Weibo had higher professionalism, which was the key factor for potential tourists to stimulate tourism intention. Thus, Experiment B would import the tourism motivation of potential tourists based on Experiment A to test the moderating effect of tourism motivation between information source and tourism intention.

### Experiment B

The purpose of Experiment B is to replicate and extend the findings of Experiment A and provide an initial test of hypotheses 3 and 4. We predicted the different impacts of tourism motivation of potential tourists on tourism intention. Considering the credibility of WeChat Moments, the extrinsic motivation of potential tourists would exert a more significant impact on tourism intentions; regarding professional tourism bloggers, the intrinsic motivation of potential tourists would exert a more significant impact on tourism intention.

#### Research design

In Experiment B, we adopted 2 (information source: credibility of WeChat Moments vs. professionalism of tourism bloggers from Weibo) × 2 (tourism motivation: intrinsic motivation vs. extrinsic motivation) research design and investigated the impacts of Daka destination information source and tourism motivation of tourists on tourism intention. The information source of Experiment B was the same as Experiment A, from the two experimental groups of WeChat Moments and tourism bloggers from Weibo, and the same form of text description and picture introduction as Experiment A was presented to the subject. In addition, the measurement of Daka destination information source credibility, professionalism, and intention tourism aligned with Experiment A. Of note, the tourism motivation scale was based on Wang et al. (2017). Furthermore, a seven-point Likert scale was adopted for all scales (1 = not at all agree and 7 = strongly agree), and subjects were asked to fill in demographic information at the end of the questionnaire.

The experiment was designed with attention verification and screening rules for continuous extreme value/continuous same value exclusion. Finally, 327 valid samples were obtained (male 52.1%; *M*_age_ = 26.64, SD 5.87). Of these, the Daka destination information source in WeChat Moments was the information published by the researcher himself in WeChat Moments. The researcher’s WeChat friends were taken as experimental objects, and 162 subjects were collected for testing. Besides the Daka destinations, information of tourism bloggers from Weibo was tested on Wenjuanxing with 165 subjects at random.

#### Results

**FIGURE 2:**
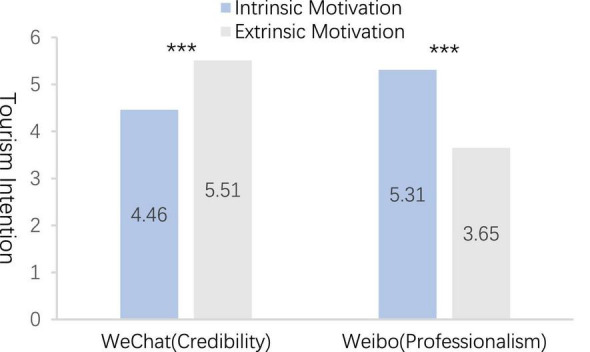
The influence of potential tourists’ motivation on tourism intention. ^***^p < 0.01.

The experimental manipulation results of Experiment B revealed that the credibility and professionalism of WeChat Moments as an information source differed significantly [*M*_credibility_ = 5.63, *M*_professionalism_ = 4.71, *F*_(1,325)_ = 61.58, *P* < 0.001]. We observed a significant difference between the credibility and professionalism of Weibo travel bloggers as information sources [*M*_credibility_ = 4.33, *M*_professionalism_ = 5.82, *F*_(1,325)_ = 49.32, *P* < 0.001], and the experimental manipulation was successful. In addition, two-way ANOVA (Figure 2) between groups demonstrated that the credibility of WeChat Moments and professionalism of tourism bloggers from Weibo had significant PEA results on potential tourists’ tourism intention [*F*_(1,323)_ = 19.83, *P* = 0.002], the main effect analysis result of tourism motivation on potential tourists’ travel intention was significant [*F*_(1,323)_ = 11.96, *P* < 0.001], and the interaction effect of the two on the main effect analysis of potential tourists’ travel intention was significant [*F*_(1,323)_ = 20.10, *P* < 0.001]. Based on the credibility of WeChat Moments as the information source, the extrinsic motivation response group had a higher potential tourists’ tourism intention than the intrinsic motivation response group [*M*_intrinsic motivation_ = 4.46, *M*_extrinsic motivation_ = 5.51, *F*_(1,163)_ = 10.24, *P* < 0.001]. Based on the professionalism of tourism bloggers from Weibo as the information source, the intrinsic motivation was higher than the extrinsic motivation reply group potential tourists’ tourism intention [*M*_intrinsic motivation_ = 5.31, *M*_extrinsic motivation_ = 3.65, *F*_(1,160)_ = 12.25, *P* < 0.001]. Thus, H3 and H4 are verified.

### Discussion

Although there have been sufficient prior studies to prove that different tourism motivations have an impact on the tourism intentions of potential tourists, the research that the tourism motivations of potential tourists combined with the stimulation of the characteristics of different social media information sources have an impact on the travel intentions of potential tourists still needs to be further verified. Therefore, based on the conclusions drawn in Experiment A, Experiment B demonstrates the influence of potential tourists’ tourism motivation and the different characteristics of different types of social media as tourism information sources on potential tourists’ tourism intentions. The empirical results of Experiment B demonstrated that Daka destinations were stimulated by different information sources, the tourism motivation of potential tourists was different, and the impacts on tourism intention were different. Based on the credibility of WeChat Moments, extrinsic motivation exerted more significant positive impacts on potential tourists’ tourism intention than the intrinsic motivation response group. Based on the professionalism of tourism bloggers from Weibo, intrinsic motivation exerted more significant positive impacts on potential tourists’ tourism intention than extrinsic motivation. In WeChat Moments, as a familiar information source, potential tourists were more likely to build reputation, meet more friends, keep in touch with friends, and get social rewards or extra rewards. The associative stimulation of extrinsic motivation made potential tourists generate tourism intention. Nevertheless, tourism bloggers from Weibo, as a strange information source, were more about intrinsic rewards and self-improvement, and focused on self-directed intrinsic motivation and associative stimulation, thereby making potential tourists generate tourism intention. Accordingly, Experiment C introduced the self-construction of potential tourists based on Experiment B to examine the moderating effect of potential tourist self-construction between information source, tourism motivation, and tourism intention in the dual context of information source and tourism motivation.

### Experiment C

The purpose of Experiment C is to replicate and extend the findings of Experiment B and provide tests of hypotheses 5 and 6. We predicted the different impacts of the self-construction of potential tourists on tourism intention. Regarding the credibility of WeChat Moments, dependent self-construction potential tourists with extrinsic motivation would exert a more significant impact on tourism intention. Regarding tourism bloggers with high professionalism, independent self-construal potential tourists with intrinsic motivation would exert a more significant impact on tourism intention.

#### Research design

In Experiment C, we adopted 2 (information source: the credibility of WeChat Moments vs. professionalism of tourism bloggers from Weibo) × 2 (tourism motivation: intrinsic motivation vs. extrinsic motivation) × 2 (self-construction: independent vs. dependent) research design. We investigated the impact of information sources, travel motivation, and self-construction of Daka destinations on potential tourists’ tourism intentions. Of note, the information source of Experiment C was the same as that of Experiment A, coming from the two experimental groups WeChat Moments and tourism bloggers from Weibo, respectively. After excluding subjects with travel experience to the case site, finalized subjects were asked to read the experimental stimulus materials and fill in the questionnaire according to their opinion.

The scale of Daka destination information source credibility, professionalism, tourism motivation, and tourism intention was the same as that in Experiment B. The self-construction measurement was based on the scale of Li (2018), and we adopted a seven-point Likert scale (1 = completely disagree and 7 = strongly agree). At the end of the questionnaire, the subject was required to fill in the demographic information. The experiment was designed with attention verification and screening rules of continuous extreme value/continuous same value exclusion. Finally, we obtained 503 valid samples (male 52.4%; *M*_age_ = 33.12, SD = 3.27). Among them, the Daka destination information source in WeChat Moments was the information published by the researcher in WeChat Moments. The researcher’s WeChat friends were taken as the experimental objects, and 267 subjects were collected for testing. Furthermore, the Daka destination information of tourism bloggers from Weibo was tested on Wenjuanxing with 236 subjects at random.

#### Results

In Experiment C, we referred to the measurement and evaluation method of self-construction proposed by Kwon and Mattila (2015). The average scores of independent self-construction and dependent self-construction items were calculated, respectively. Dependent self-construction was subtracted from independent self-construction, and the subject’s self-construction score was obtained. While subjects with positive scores were divided into independent self-construction groups, those with negative scores were divided into dependent self-construction groups. Among them, 281 people were assigned to the independent self-construction response group and 222 to the dependent self-construction response group.

The experimental manipulation results of Experiment C revealed that the credibility and professionalism of WeChat Moments as an information source differed significantly [*M*_credibility_ = 5.86, *M*_professionalism_ = 4.71, *F*_(1,265)_ = 33.15, *P* < 0.050], and the credibility and professionalism of tourism bloggers from Weibo as an information source were significant [*M*_credibility_ = 4.17, *M*_professionalism_ = 5.62, *F*_(1,234)_ = 19.22, *P* < 0.001]. In the stimulation situation of WeChat Moments information source, extrinsic motivation was higher than the potential tourists’ tourism intention in the intrinsic motivation response group [*M*_intrinsic motivation_ = 4.41, *M*_extrinsic motivation_ = 4.96, *F*_(1,499)_ = 24.56, *P* < 0.001]. In the stimulation situation of tourism bloggers from the Weibo information source, the intrinsic motivation response group had a higher potential tourists’ tourism intention than the extrinsic motivation response group [*M*_intrinsic motivation_ = 5.02, *M*_extrinsic motivation_ = 3.73, *F*_(1,499)_ = 28.14, *P* < 0.001]. Furthermore, the interaction effect was significant [*F*_(1,499)_ = 29.04, *P* < 0.001], and the experimental manipulation was successful.

**FIGURE 3:**
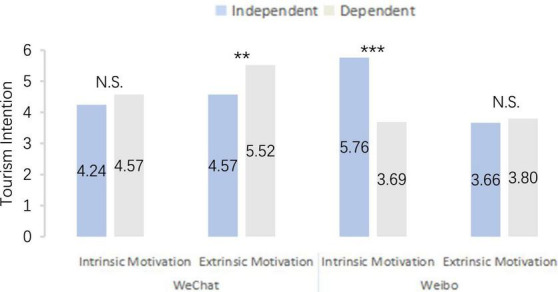
The influence of potential tourists self-construction on tourism intention. ^***^p < 0.01, ^**^p < 0.05. N.S. = no significant.

The between-group multivariate ANOVA (Figure 3) revealed that the credibility of WeChat Moments and professionalism of tourism bloggers from Weibo had a significant PEA result on potential tourists’ tourism intention [*F*_(1,494)_ = 10.70, *P* < 0.001]. For potential tourists’ tourism intention, the PEA result of tourism motivation was significant [*F*_(1,494)_ = 11.37, *P* = 0.001], the PEA result of self-construction was not significant [*F*_(1,494)_ = 1.08, *P* = 293], and the interaction effect of the three was significant on the PEA results [*F*_(1,494)_ = 7.03, *P* = 0.008]. Under the stimulation situation of WeChat Moments information source, the potential tourists’ tourism intention with extrinsic motivation based on dependent self-construction was higher [*M*_independent_ = 4.57, *M*_dependent_ = 5.52, *F*_(1,123)_ = 14.77, *P* < 0.05]. No significant difference was noted between the groups of potential tourists with intrinsic motivation based on independent self-construction and dependent self-construction [*M*_independent_ = 4.24, *M*_dependent_ = 4.57, *F*_(1,140)_ = 1.27, *P* = 0.163]. In addition, tourism bloggers from Weibo information source had higher potential tourists’ tourism intention with intrinsic motivation based on independent self-construction [*M*_independent_ = 5.76, *M*_dependent_ = 3.69, *F*_(1,123)_ = 35.53, *P* < 0.001]. We observed no significant difference between the groups of potential tourists holding extrinsic motivation based on independent self-construction and dependent self-construction [*M*_independent_ = 3.66, *M*_dependent_ = 3.80, *F*_(1,109)_ = 6.31, *P* = 0.728]. Hence, H5 and H6 are verified.

#### Discussion

Although previous studies have proved that the self-construction of potential tourists will have an impact on tourism intention, the specific research on the stimulation of different social media information sources and the impact of tourism motivation on potential tourists’ tourism intention needs to be supplemented and verified. Therefore, based on the theory verified by Experiment A and Experiment B, the regulating effect of potential tourists’ self-construction on tourism intention under different Daka destination information sources and tourism motivation scenarios was tested in Experiment C. Based on the theory verified by Experiment A and Experiment B, the regulating effect of potential tourists’ self-construction on tourism intention under different Daka destination information source and tourism motivation scenarios was tested in Experiment C. The results aligned with the hypothesis; based on the credibility of WeChat Moments, potential tourists with extrinsic motivation, the dependent self-construction response group had more significant positive impacts on tourism intention than the independent self-construction response group. However, no significant difference was noted between independent self-construction and dependent self-construction response groups for potential tourists with intrinsic motivation. Based on the professionalism of tourism bloggers from Weibo, potential tourists with intrinsic motivation, and independent self-construction exerted more significant positive impacts on tourism intention than the dependent self-construction reply group. In contrast, potential tourists with extrinsic motivation, independent self-construction, and dependent self-construction responses did not differ markedly between groups.

## Conclusion and inspirations

The following are the conclusion and research limitations and prospects.

### Conclusion

With the development of social media and the extensive popularity of audio-visual users, Daka destinations developed based on social media platforms have become the main choice for potential tourists in the post-pandemic era. From the construction theory perspective, this study explored the impacts of Daka destination information source on tourism intention, providing the empirical theoretical basis for tourism recovery in the post-pandemic era. The conclusions drawn are as follows:

(1) Different information sources had different stimulations on potential tourists. WeChat Moments positively affected potential tourists’ tourism intention based on credibility. In addition, tourism bloggers from Weibo exerted more significant positive impacts on potential tourists’ tourism intention based on professionalism. That is, different tourism attraction information sources had different impacts on the travel decisions of potential tourists because the Daka destination information shared in WeChat Moments focused more on trust among friends, while that shared by tourism bloggers on Weibo Tourism information needed to focus more on professional knowledge.
(2) In the context of tourism information stimulation, the impacts on tourism intention would differ based on the tourism motivation of potential tourists. In the context of WeChat Moments credibility, the extrinsic motivation of potential tourists exerted a more significant impact on tourism intention. From the perspective of the professionalism of tourism bloggers from Weibo, the intrinsic motivation of potential tourists exerted a more significant impact on tourism intention.
(3) In the dual context of information source and tourism motivation, based on the different self-construction of potential tourists, the impacts on tourism intention also differed, and interaction occurred between the credibility and professionalism of information source, tourism, and the self-construction of potential tourists. From the perspective of WeChat Moments credibility, dependent self-construction potential tourists with extrinsic motivation exerted more significant impacts on tourism intention. Nevertheless, no significant difference was found in potential tourists with intrinsic motivation. That is, potential tourists with dependent self-construction with extrinsic motivation acquire and accept Daka destination information based on the trust of WeChat Moments to stimulate tourism intentions. Besides, no significant difference was observed in potential tourists with extrinsic motivation. That is, potential tourists with dependent self-construction with extrinsic motivation acquire and accept Daka destination information based on the trust of WeChat Moments to stimulate tourism intentions. Furthermore, potential tourists with independent self-construction with intrinsic motivation will generate tourism intention based on the professionalism of tourism bloggers from Weibo to attain and accept Daka destination information.

The management implications of this study are as follows:

(1) The utility of social media information largely depends on the perceived professionalism of the information source and the receiver’s trust in the information source (Tham et al., 2013). The lack of social clues to social media information sources will have a negative impact on the credibility of online comments (Ye et al., 2009). The professional level of information sources has different effects on the certainty of consumer attitudes (Tormala and Petty, 2004). WeChat Moments, a social media considered being a real interpersonal relationship, should be more inclined to the credibility of information publishing. Moreover, tourism bloggers from Weibo should have professional knowledge and colorful experiences to provide tourists with a full range of travel information. Information processing theory can explain this phenomenon which treats the consumer’s decision-making process as a process of processing information, which requires consumers to process vast amounts of information and incur cognitive effort and cost (Salanick and Pfeffer, 1978). When consumers lack the ability to judge and evaluate travel information, it often means that more time and cognitive effort are needed to process travel-related information. Furthermore, this study expands the research field of Daka destinations tourism intention from the initial stage of tourism information source, providing a more effective theoretical basis for Daka destination information marketing.
(2) In the era of social media, potential tourists can receive information from multiple channels, yet different information sources produce different stimulating effects. In this study, potential tourists with intrinsic motivation involved a high degree of autonomy and tended to be proactive, willing to explore and travel for their pleasure and satisfaction. Moreover, potential tourists with extrinsic motivation involved low autonomy, motivated by extrinsic factors that forced individuals to display behaviors that pursued favorable outcomes or rewards. Regarding different tourism information sources, tourism motivation of potential tourists was also a key factor affecting travel decisions of potential tourists. The results extended the analysis and evaluation model of different tourism information channels under multidimensional complex relationships. Besides, the formation mechanism of tourist tourism intention in the Internet era provided reference and guidance for further research.
(3) This study combined the self-construction theory with information source features and tourism motivation and examined the dynamic changes of independent and dependent self-construction in the process of information source adoption and processing from the standpoint of potential tourists. Potential tourists guided by independent self-construction orientation focused more on their inner calling and wishes when making travel decisions and were less susceptible to impacts from external environmental factors. Nevertheless, potential tourists guided by dependent self-construction orientation were subject to the impact of group norms, valued the value of group harmony, and exhibited convergence features with their behaviors. This study enriches the research mechanism of the formation path of potential tourists’ tourism intention, extends the self-construction theory to the research field of using social media to collect Internet celebrity tourism information, and provides a reference for subsequent research on potential tourists’ tourism intention.

### Research limitations and prospects

Owing to the limitations of the experimental conditions, this study’s results could still be refined further by follow-up research. First, this study adopted the social media WeChat Moments and tourism bloggers from Weibo, used commonly in daily life, as two different information sources. Although the relevant hypotheses were validated in this study, the research conclusions were not universal, and the explanatory power of the overall social media industry was limited. Thus, other social media should be chosen for further verification. Second, tourism motivation of potential tourists and self-construction were examined per experimental stimulus, and the impacts of different tourism information sources on tourism intention were assessed. There could be other more crucial factors that influenced potential tourists’ tourism intention, necessitating follow-up studies. Finally, Wenjuanxing was used to collect unfamiliar groups where tourism bloggers from Weibo were used as the experimental sample of the information source. Although the external validity was satisfied, the homogeneity of the samples could not be satisfied. Hence, subsequent research could use the same sample group for further corroboration.

## Data availability statement

The raw data supporting the conclusions of this article will be made available by the authors, without undue reservation.

## Author contributions

YC, JZ, and SY contributed to conception and design of the study. LW organized the database. YC and JD performed the statistical analysis and wrote the first draft of the manuscript. YC and JZ wrote sections of the manuscript. All authors contributed to manuscript revision, read, and approved the submitted version.

## Footnotes

1 For the image of the WeChat moments see https://www.wjx.cn/vm/motX8T3.aspx. For the image of the Weibo post see https://www.wjx.cn/vm/QVXNAZl.aspx.
